# Behcet's Disease: Systemic and Ocular Manifestations

**DOI:** 10.1155/2013/247345

**Published:** 2013-10-03

**Authors:** Jelena Paovic, Predrag Paovic, Vojislav Sredovic

**Affiliations:** ^1^Department of Ophthalmology, Clinical Centre of Belgrade, Pasterova 2, Belgrade 11000, Serbia; ^2^Uvea Center, Center for Diagnostic and Treatment of Uveitis, Kneza od Semberije 14, Belgrade 11000, Serbia

## Abstract

*Aim*. The aim of this study was to evaluate if patients with Behcet's disease who have ocular involvement have a more severe form of this disease as compared to patients with Behcet's disease alone. *Methods*. A total of 99 patients were included in the study. 76 patients were used as part of the examined group, and 23 patients formed a control group. *Results*. The following are the results of examined and control groups, respectively: recurrent oral aphthous ulcers 89.5%, 95.7%; genital ulcers 61.8%, 97.0%; articular involvement 72.4%, 65.2%; vasculitis 81.6%, 60.9%; positive pathergy test 25.0%, 47.8%. Higher frequency of genital ulcerations was noted in control group (*P* = 0.001). More than two major criteria were met in 100% of the cases. HLA B51 was present in 78.9% of the cases in the examined group and 43.5% of the cases in control group; thus there is significant difference between them (*P* = 0.001). Visual acuity >0.5 occurred in 76% (examined group). Most frequent ocular manifestations in the examined group were retinal periphlebitis 81.6%, periphlebitis and periarteritis 65%, and serofibrinous uveitis 63.2%. Macular edema as a complication was present in 63.2%. The majority of patients (55.3%) were treated with combined therapy consisting of cyclosporine A and systemic corticosteroids. In 38.2% of patients, laser photocoagulation was used on retinal periphery.

## 1. Introduction

Behcet's disease is an autoimmune, rare, and severe multisystemic inflammatory disease characterized by recurrent oral aphthous ulcers, genital ulcers, skin lesions, and both anterior and posterior uveitis. 

The first series of patients with Behcet's disease was published in 1937 as a triad of symptoms consisting of oral aphthous ulcers, genital ulcers, and hypopyon iritis [1]. 

International Study Group for Behcet's Disease (ICBD) established diagnostic criteria for Behcet's disease; ocular lesions, oral aphthous ulcerations, and genital aphthous ulcerations are each assigned 2 points, while skin lesions, central nervous system involvement, and vascular manifestations are assigned 1 point each. The pathergy test, when used, was assigned 1 point. A patient scoring ≥4 points is classified as having Behcet's disease [2]. Disease is characterized by episodic inflammations which may affect every tissue and organ of the body: joints, gastrointestinal tract, nervous system, and others. Generally, Behcet's disease is a secondary occlusive systemic vasculitis, which affects both arteries and veins of all sizes and tissue types [3–12].

The prevalence of the disease is much higher in countries bordering the “Old Silk Route.” There are many reports on clinical manifestations of Behcet's disease from different parts of the world such as Turkey, Iran, Japan, China, and England. Each of the studies was done on a great number of patients, 3443 Iran [13]; 2806 Iran [14]; 2313 Turkey [15]; 2147 Turkey [16]; 880 Turkey [17]; 3316 Japan [18, 19]; 260 Tunis [20]; 419 England [21]. Other series are based on a sample smaller than 200 patients [22]. 

Demographic characteristics, clinical features, and famillial occurrence of the disease observed in the groups differ due to environmental and/genetic factors [23].

Amongst various genetic markers, class I, HLA-B5, and its subclass B51 allele have the highest reported association with Behcet's disease. The highest susceptibility is present in individuals living in areas along the Silk Route [24–33]. 

Ocular involvement as a result of irreversible, progressive, ischemic damage of the retina, and optic disc, commonly leads to severe panuveitis and its complications [34–38].

Corticosteroid therapy and cytostatic drugs (combined or not) were commonly used depending on the severity and morbidity of the disease [38–45]. New therapy, such as anti-TNF-*α*, represents some of the positive achievements which have been made in this field [45–53].

## 2. Methods 

Systemic manifestations of Behcet's disease have been categorized into two groups: examined group of 76 patients with ocular manifestations and control group of 23 patients without ocular manifestations. All patients met the classification criteria of the International Study Group for Behcet's Disease. Information on patients' gender, age, systemic manifestations, ocular features and various complications, visual acuity, and systemic treatment were analysed.

## 3. Results 

The examined group which was part of the research sample of 76 patients in total was taken under consideration, comprising 43 (56.6%) males and 33 (43.4%) females.

There was no noted significant difference in prevalence (*P* = 0.138, *P* > 0.05), and further assessment does not take patients gender into consideration as it holds no statistical bearing for this analysis. 

There was noted significant difference in prevalence between gender in the control group of 23 patients (males 60.9% and females 39.1%). 

Average age of both males (31.7%) and females (32.3%) was approximately the same (around 32 years) and held no statistical significance in the examined and control groups. Behcet's disease is manifested familially in 4 cases (two brothers and a brother and a sister) in the examined group. There was HLA-B51 present between all siblings. 

Table 1 depicts systemic manifestations of Behcet's disease (categorized in two groups: examined and control), from which it can be noted that the majority of individuals (examined 89.5% and control 95.7%) had recurrent oral ulcers.

Second the most frequent systemic manifestation in patients with Behcet's disease was articular involvement (which was noted to have occurred in a number of cases but had no statistical significance when compared to the control group (*P* = 0.294)).

Higher frequency of genital ulcerations was noted in the control group as compared to the examined group (*P* = 0.001, *P* < 0.05). 

Vascular diseases were present in a substantial number of cases (81.6% versus 60.9%, *P* = 0.04), while cutaneous lesions did not differ significantly between groups (*P* = 0.144). There are significantly smaller frequency and difference of various other systemic manifestations (neurological changes, digestive tract ulcerations, and thrombophlebitis), in both the control and the examined groups of patients (Table 1).

Research sample contained 89.5% and 95.7% of patients with recurrent oral ulcerations in the examined and control groups, respectively. All patients had more than 4 points (ISBD) (Table 2).

HLA-B51 was present in 78.9% of the cases in the examined group and 43.5% of the cases in control group; thus there is significant difference between them (*P* = 0.001). Besides HLA-B51, two patients from the examined group were also positive for HLA-B27, marker for seronegative arthropathy (Table 3).

Table 4 shows visual acuity >0.5, noted in a sample of 76 patients, as having been around 64% right eye and around 55% left eye. Table 4 also shows visual acuity <0.1 noted in a significantly smaller sample of patients (i.e., between 0.1 and 0.5).

**Figure 1:**
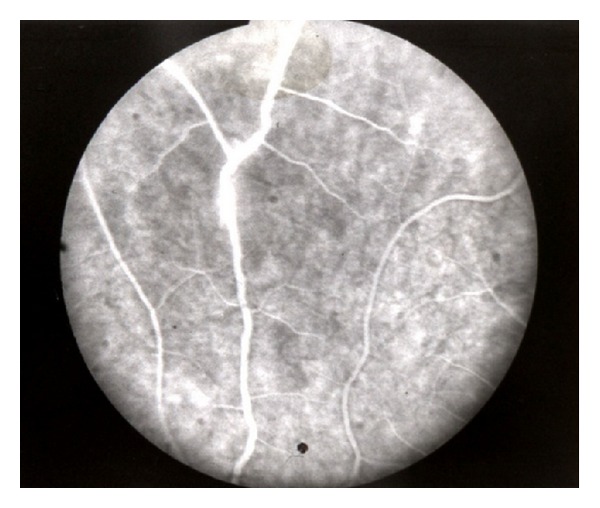
Behcet's disease, retinal periphlebitis.

Ocular manifestations in patients with Behcet's disease were present on both the anterior and posterior segments of the eye (Table 5). Most frequent ocular manifestation was retinal periphlebitis, followed by periphlebitis together with inflammation of arteries (81.6% versus 65.0% at *P* = 0.03 < 0.05) (Table 5, Figure 1).

Subsequently, the next most frequent ocular manifestation was serofibrinous iridocyclitis (63.2%) then cyclitis in around 38% (significantly less frequent occurrence, *P* = 0.02). Fibrinopurulent iridocyclitis (presence of hypopyon in anterior chamber) was diagnosed in 19.7% of the cases. Papillitis was seen in 16 (21.5%) of the cases (Table 5).

Allocation of patients according to severity of inflammatory processes in Behcet's disease is shown in Table 6. 

Severe form of the disease was noted in approximately 71% of the cases, a significantly higher incidence in comparison to other degrees of diseases (*P* = 0.0006, *P* ≤ 0.001). Moderate form of the disease was present in approximately 1/3 of patients, while mild form of this disease was rarely observed (Table 6).

**Figure 2:**
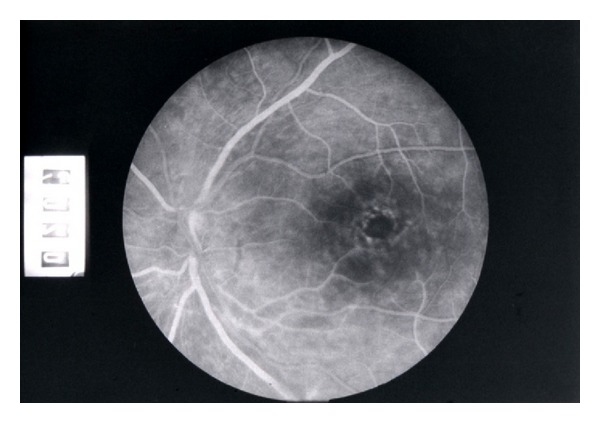
Macular edema in patients with uveitis.

Complications of uveitis on the anterior segment of the eye were as follows: complicated cataract 39.5% and secondary glaucoma 17.1%. According to frequency of occurrence, these two complications did not differ significantly from one another (*P* = 0.07). Most common complication of the posterior segment of the eye was macular edema 63.2% (Figure 2), whilst optic disc edema and optic disc atrophy were equally represented (around 21% *P* = 0.001) (Table 7). 

Table 8 shows treatment of uveitis in Behcet's disease.

Decision on treatment type and drug dosages was made during the initial examination and depended on the severity of the disease. 

Systemic corticosteroid therapy was implemented in 30.3% of patients.

The majority of patients (55.3%) were treated with combined therapy consisting of cyclosporine A and systemic corticosteroids. 

Daily dosages of the above mentioned agents were as follows: 4-5 mg/kg of cyclosporine A daily and 20 mg of corticosteroids (prednisolone) daily.

Patient's liver and kidney functions, as well as concentrations of cyclosporine A in blood, were regularly monitored. 

Cytostatic drugs: azathioprine (50–150 mg/day), cyclophosphamide (2 mg/kg daily), methotrexate (7.5–25 mg single dose, once a week), were given to 14.5% of patients. 

In 38.2% of patients, laser photocoagulation (PHC) was used on retinal periphery, around blood vessels, in areas of retinal ischemia, around various tears, and retinoschisis, as well as in places where retinal thinning had occurred.

In 1/3 of patients, laser PHC was combined with repeated sub-Tenon's injections of triamcinolone acetonide (20 mg). Therapy generally consisted of 4–6 doses.

**Figure 3:**
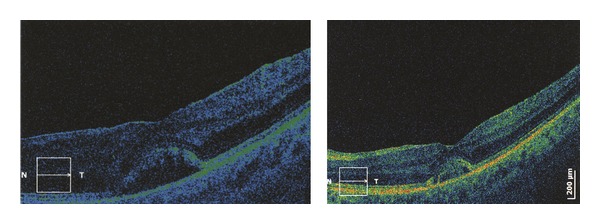
Macular edema in patients with uveitis, OCT findings (followup).

Evolution of macular edema was monitored via optical coherent tomography (OCT) (Figure 3).

**Figure 4:**
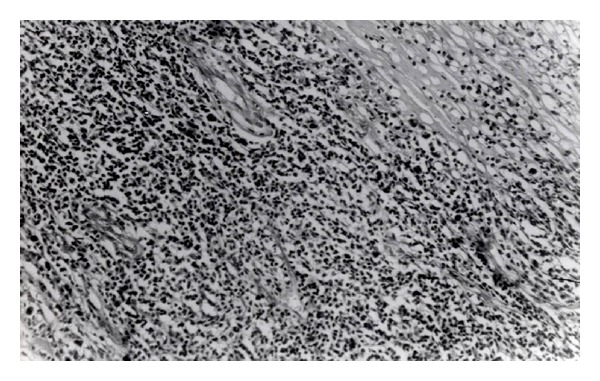
Behcet's disease, historical findings.

Uveitic complications have been surgically treated as follows: cataract operation in 6.8% of cases; combined phacoemulsification and vitrectomy in 5.3% of cases; glaucoma surgery in 5.3% of cases; and in one case (1.3%) enucleation being performed (Table 8) (Figure 4).

Histological findings were as follows: obliterative perivasculitis (periphlebitis) and venous thrombosis with lymphocytic and monocytic cellular infiltration in veins, capillaries, and arteries.

## 4. Discussion

Systemic manifestations of Behcet's disease were examined in 76 patients with ocular manifestations of the disease (examined group) and 23 patients (control group) without ocular manifestations. Gender distribution between groups (examined and control) was noted. Male to female ratio was 0.98 in Japan, 0.63 in Korea, 1.19 in Iran, 1.03 in Turkey, and 1.8 in India [5].

Average age of both males (31.7) and females (32.3) was approximately the same (around 32 years) and had no statistical significance between groups. Different reports stated that there was smaller variability in ages at the onset of the disease: 35.7 in Japan, 29 in Korea, 26 in Iran, 25.6 in Turkey, 29 in Greece, 24.5 in Germany, and 24.7 in UK [5].

In our series clinical features of Behcet's disease in examined and control groups were significantly dominated by mucous membrane manifestations (oral and genital aphthous ulcerations). Oral aphthous ulcerations were predominantly in the examined group (89.5%), and genital ulcers were dominant in the control group (97.0%).

Oral aphthous ulcers were seen in 96.8% of patients in Iran, 98.2% in Japan, 100% in Turkey, 97.5% in Korea, 100% in Morocco, and 100% in England [5]. Genital aphthous ulcerations were seen less frequently. They were detected as follows: in 65.3% in Iran, 73.2% in Japan, 88.2% in Turkey, 56.7% in Korea, 83.5% in Morocco, and 89% in England [5]. 

The third systemic manifestation in both groups was vascular involvement, which occurred more frequently, but still had no statistical significance, in the examined group (81.6%) as compared to the control group (60.9%). 

Articular involvement was seen in 34% in Iran, 57% in Japan, 16% in Turkey, 24% in Korea, 56.9% in Morocco, and 93% in England [5].

In our series joint involvement was present in 72.4% (examined group) and in 65.2% (control group).

There was no noted difference between examined and control groups. 

Skin lesions were present in 55.3% of the examined group and 34.8% of the control group. There was no noted difference between groups. Skin lesions in form of pseudofolliculitis were commonly seen.

Skin lesions were also seen in 69.3% in Iran, 87.1% in Japan, 60.6% in Korea, and 86.3% in England [5].

Other manifestations such as neurological and digestive tract ulcerations were less present. 

By comparing our results with results of other series, it can be seen that there exists small variation depending on an association of environmental factors together with histocompatibility antigen.

Pathergy test was often present in the control group (47.8%), as opposed to the examined group (25.0%), but was not statistically significant. In our series there exists significant difference in HLA-B51 between two groups (control 43.5% versus examined 78.9%). 

Eye is the most commonly involved organ in Behcet's disease, affected within 2–4 years of its onset. There exist cases of Behcet's disease without ocular manifestations, which have been registered in our control group. Generally, initial inflammatory ocular process is more anterior and unilateral and later on tends to involve the posterior segment of the eye becoming bilateral. In the majority of cases, it is present as panuveitis.

In our series retinal periphlebitis was the most common ocular manifestation 81.6%, following which was retinal periphlebitis associated with periarteritis 65.0%. Anterior segment inflammation as serofibrinous iridocyclitis was present in 63.2% and so was more frequent as compared to other series where fibrinopurulent iridocyclitis had been dominant [35–37]. 

In our study severe form of uveitis was dominant (71.1%).

Visual loss may also develop as a result of retinal vasculitis and its complications, such as macular edema and others.

Cystoid macular edema was the most common complication in the examined group (63.2%). Cataract and glaucoma were frequent ocular complications of uveitis. In our series, complicated cataract was diagnosed in 63.2% of individuals and phacoemulsification was performed.

Typically, patients have episodes of severe uveitis and retinal vasculitis that progressively damaged vision.

Systemic corticosteroids and/cytostatic agents are needed [38–45]. 

In our series cyclosporine A and systemic corticosteroids were used in 55.3%.

Generally, patients tolerated medication well. However, retinal and liver toxicity to cyclosporine A therapy prevented prescription of a maximally affective therapeutic dose of the drug. Cyclosporine A levels in plasma were dose dependent for each patient. New therapeutic procedures, such as biological therapy, anti-TNF*α* agents, have been used over the last couple of years [46–53].

Seeing that the majority of patients had a clinical manifestation of retinal vasculitis, whose most common complication was macular edema, laser PHC was applied to retinal periphery and around blood vessels, as well as in places around peripheral retinal degeneration (38.2% of cases).

Additional therapy: due to macular edema repeated doses of subtenon's triamcinolone acetonide injections were given. In cases with severe forms of panuveitis, cytostatic therapy was used. Histological studies, which had aimed to prove changes in tissues such as papulopustular lesions and lesions in ocular tissue, have been previously performed [54, 55]. Histological finding in our patient showed that the primary change is vasculitis. 

## 5. Conclusion 

The most common systemic manifestations in both groups were as follows: oral aphthous ulcerations, genital ulcerations, vascular manifestations and joint disorders. All patients in control and examinated groups had more than 4 points. Group of patients which had no ocular manifestations had genital ulcerations which occurred more frequently and were statistically significant. Those patients which were positive for HLA-B51 had high frequency of ocular manifestations. 

The most common ocular manifestation was retinal vasculitis. Cystoid macular edema was the most common ocular complication of retinal vasculitis and at the same time the main reason for decrease in visual acuity. 

Besides systemic therapy, due to ocular complications, subtenonial application of triamcinolone acetonide injections and laser PHC on the retinal periphery is recommended. 

## Figures and Tables

**Table 1:** Behcet's disease, systemic manifestations.

Systemic manifestations	Examined group (N = 76)		Control group (N = 23)	
	n	%	n	%
Recurrent oral aphthous ulcers	68	**89.5**	22	**95.7**
Genital ulcers	31	**61.8**	20	**97.0**
Articular involvement	55	**72.4**	15	**65.2**
Digestive tract ulcerations	3	13.2	5	21.7
Cutaneous lesions	42	55.3	8	34.8
Vascular diseases	62	**81.6**	14	**60.9**
Thrombophlebitis	3	3.9	4	17.4
Neurological diseases	12	15.8	3	13.0

**Table 2:** ISBD criteria for diagnosing Behcet's disease.

Complete disease	Examined group (N = 76)		Control group (N = 23)	
	*n*	*% *	*n*	*% *
Recurrent oral ulcerations	68	**89.5**	22	**95.7**
*Plus any 2 of the following *				
Recurrent genital ulcerations	31	40.8	20	**87.0**
Ocular lesions	76	100.0	0	0.0
Skin lesions	42	55.3	8	34.8
Positive pathergy test	19	25.0	11	47.8

**Table 3:** Behcet's disease, HLA typing.

HLA	Examined group (N = 76)		Control group (N = 23)	
	n	%	N	%
HLA-B51	60	78.9	10	43.5
HLA-B51 and HLA-B27	2	2.6	0	00.0

**Table 4:** Behcet's disease, visual acuity of patients with uveitis.

Visual acuity	VOD		VOS	
	n	%	N	%
<0.1	11	14.5	22	28.9
0.1–0.5	16	21.0	12	15.8
>0.5	49	64.5	42	55.3
Total	**76**	**100**	**76**	**100**

**Table 5:** Behcet's disease, ocular manifestations.

Ocular manifestations	Examined group (*N* = 76)	
	*n*	%
*Anterior uveitis *		
Serofibrinous iridocyclitis	48	63.2
Fibrinopurulent iridocyclitis	15	19.7
Cyclitis	29	38.2
*Retinal blood vessel involvement *		
Retinal periphlebitis	62	81.6
Periphlebitis and periarteritis	39	65.0
Papillitis	16	21.5

**Table 6:** Behcet's disease, intensity of uveitic processes.

Intensity of processes	Examined group (N = 76)	
	*n*	*% *
Severe	54	71.1
Moderate	18	33.7
Mild	4	5.2
Total	**76**	**100**

**Table 7:** Behcet's disease, complications of uveitis.

Complications	Examined group (N = 76)	
	N	%
*Complications: anterior segment of the eye *		
Cataract	30	39.5
Secondary glaucoma	13	17.1
*Complications: posterior segment of the eye *		
Macular edema	48	63.2
Disc edema	16	21.1
Disc atrophy	16	21.1

**Table 8:** Behcet's disease, treatment of uveitis.

Treatment of uveitis	Examined group (N = 76)	
	*n*	*% *
*Medical and/laser PHC treatment *		
Corticosteroid drops	76	100.0
Dexasone subconjunctival	16	21.1
Systemic corticosteroids	23	30.3
Cyclosporine A and corticosteroids	42	55.3
Cytostatic drugs	11	14.5
Laser PHC	29	38.2
Laser PHC and triamcinolone acetonide	11	14.5
*Surgical treatment *		
Phacoemulsification	9	6.8
Phacoemulsification and vitrectomy	4	5.3
Antiglaucoma surgery	4	5.3
Enucleation	1	1.3
